# GC–MS Analysis, Antioxidant and Antimicrobial Activities of *Achillea Odorata* Subsp. *Pectinata* and *Ruta Montana* Essential Oils and Their Potential Use as Food Preservatives

**DOI:** 10.3390/foods9050668

**Published:** 2020-05-22

**Authors:** Taoufiq Benali, Khaoula Habbadi, Abdelmajid Khabbach, Ilias Marmouzi, Gokhan Zengin, Abdelhakim Bouyahya, Imane Chamkhi, Houda Chtibi, Tarik Aanniz, El Hassan Achbani, Khalil Hammani

**Affiliations:** 1 Laboratory of Natural Resources and Environment, Polydisciplinary Faculty of Taza, Sidi Mohamed Ben Abdellah University of Fez, B.P. 1223 Taza-Gare, Taza, Morocco; chtibi.houda19@gmail.com (H.C.); khalil.hammani@usmba.ac.ma (K.H.); 2 Laboratoire de recherche et de protection des plantes URPP-INRA-Meknès, 50000 Meknès, Morocco; khaoula405@gmail.com (K.H.); achbanieh@gmail.com (E.H.A.); 3 Laboratory of materials, natural substances, Environment and Modeling (LMSNEM), Polydisciplinary Faculty of Taza, Sidi Mohamed Ben Abdellah University of Fez, B.P. 1223 Taza-Gare, Taza, Morocco; khamajid@hotmail.com; 4 Laboratory of de Pharmacology et Toxicology, Faculty of Medicine and Pharmacy, University Mohammed V in Rabat, BP 6203, Rabat Instituts, Rabat, 6203 Rabat, Morocco; ilias.marmouzi@gmail.com; 5 Biochemistry and Physiology Laboratory, Faculty of Science, Department of Biology, Selcuk University, 42130 Konya, Turkey; gokhanzengin@selcuk.edu.tr; 6 Laboratory of Human Pathologies Biology, Faculty of Sciences, and Genomic Center of Human Pathologies, Faculty of Medicine and Pharmacy, Department of Biology, Mohammed V University in Rabat, 1014 Rabat, Morocco; boyahyaa-90@hotmail.fr; 7 Microbiology and Molecular Biology Team, Center of Plant and Microbial Biotechnology, Biodiversity and Environment, Faculty of Sciences, Mohammed V University, Rabat, 1014 Rabat, Morocco; chamkhi.imane@gmail.com; 8 Medical Biotechnology Laboratory (MedBiotech), Rabat Medical & Pharmacy School, Mohammed V University in Rabat, 6203 Rabat, Morocco; tarik.aanniz@gmail.com

**Keywords:** *Achillea odorata* subsp. *pectinata*, Ruta montana, essential oil, antimicrobial activity, model food system

## Abstract

In order to discover new natural resources with biological properties, the chemical composition, the antioxidant and antimicrobial activities, and the potential use as food preservative of essential oils of Moroccan *Achillea odorata* subsp. *pectinata* (AO_p_EO) and *Ruta montana* (RMEO) were studied. Gas chromatography-mass spectrometry (GC-MS) analysis revealed the presence of 21 and 25 compounds in AO_p_EO and RMEO, respectively. The results showed that the major compounds of AO_p_EO are camphor (45.01%), bornyl acetate (15.07%), borneol (11.33%), *β*-eudesmol (4.74%), camphene (3.58%), and 1.8-cineole (eucalyptol) (2.96%), whereas 2-undecanone (63.97%), camphor (3.82%) and cyclopropanecarboxylic acid (3.66%) were the main components of RMEO. The antioxidant activities were evaluated by diphenylpicrylhydraziyl radical (DPPH) and reducing power assays. The antimicrobial activities of essential oils were tested against bacterial strains and food contaminant yeast using agar disc diffusion and microdilution methods. A significant antimicrobial activity of AO_p_EO was observed against *Bacillus subtilis*, *Proteus mirabilis* and *Candida albicans*, compared to RMEO. The efficacy of AO_p_EO was also evaluated in model food systems (cabbage and barley) artificially inoculated during storage. The results found that the adding of a minimal inhibitory concentration (MIC) and 4× MIC were potent in decreasing the *Proteus mirabilis* growth in food model systems. Our findings suggested that AO_p_EO may be potentially used as an alternative food preservative.

## 1. Introduction

Microbial attack causes serious loss in the organoleptic and health qualities of food products worldwide, and this problem represents a major challenge for food industry [1]. In this regard, ensuring food safety, while meeting demands for the retention of their nutritional value and quality, is an important international challenge [2]. The most used strategy to overcome the undesirable microorganism activities is the use of chemical products that possess antimicrobial and antioxidant effects, with potential side effects on the consumer [3,4,5]. This has led not only to the reduction of some chemicals but also to the restriction of some others. Recently, consumer exigency for natural molecules as an alternative preservative to replace chemical products is increasing [6,7,8]. Among the secondary metabolites, the natural mixtures of volatile and hydrophobic compounds produced by medicinal and aromatic plants—namely, essential oils (EOs)—have been widely used for a long time in medicine, perfumery and cosmetics, and were added as spices or herbs in food preparations [9,10]. In recent years, the essential oils have received increasing attention as they exhibit significant antibacterial, antifungal, and antioxidant properties [11,12,13,14,15,16]. Moreover, the effectiveness of EOs as food preservatives reported by many studies are encouraging [8,17,18,19,20,21]. Some compounds occurring in essential oils, such as thymol, cinnamaldehyde, limonene, camphor, carvacrol, borneol, linalool, terpineol-4-ol, and 1,8-cineole, are considered effective natural antimicrobial agents against foodborne strains [22,23,24,25,26,27,28,29,30]. For these proprieties, carvacrol, cinnamaldehyde, limonene, and thymol have been accepted by the European Commission and the United States Food and Drug Administration (FDA) for use as flavorings in foodstuffs, since they are considered to present no risk to the health of the consumer [4,9].

*Achillea odorata* (subspecies unspecified), a member of *Asteraceae* family, has been used as an anti-inflammatory [31], anti-diabetic [32], anti-rum, stimulating tonic, and for ethno-veterinary treatments (cattle, poultry and dogs) [33,34]. The *Achillea* species is largely distributed throughout North America, different parts of Europe, the Mediterranean regions, Eastern and Western Asia, Australia, New Zealand and the Middle East regions [35,36]. Previous papers reported the antimicrobial and antioxidant properties [37,38,39,40] of the essential oils of the *Achillea* species; thus, the chemical composition of the essential oils of all species is characterized by high amounts of oxygenated monoterpenes, specifically 1,8-cineole camphor and borneol [41], with promising antimicrobial and antioxidant activities [42,43].

*Ruta montana*, a herb from the family *Rutaceae*, is a commonly used plant in traditional medicine to treat diabetes mellitus, in abscesses treatment, an emetic in pediatric treatment, and in treating psychic sicknesses [33,34,44]. The main habitats of the *Ruta* species are concentrated in the Mediterranean region [45], and grow widely in tropical and temperate countries [46]. Many studies have reported the antimicrobial and antioxidant activities that essential oils of the *Ruta* species exhibit [47,48,49,50]. The volatile variation of the *Ruta* species’ essential oils is known by the abundance of 2-Undecanone [47,51,52,53,54]; this ketone was reported as a promising antifungal and antibacterial compound [50,55].

To the best of our knowledge, no reports on the variation of essential oil composition, or the antioxidant and antimicrobial activities, of *Achillea odorata* subsp. *pectinata* and *Ruta montana* collected from the Province of Taza, Northern Morocco, are available; although several studies exist with respect to the in vitro antimicrobial properties of essential oils, just a few investigations into their activity in food systems have been reported in the literature. Therefore, the purpose of this work is to determine the chemical composition of AO_p_EO and RMEO, to evaluate their antioxidant and antimicrobial effects, and to examine for the first time the efficacy of AO_p_EO in preserving cabbage and barley model food systems during storage.

## 2. Materials and Methods

### 2.1. Plant Material and Isolation of Essential Oils

**Figure 1:**
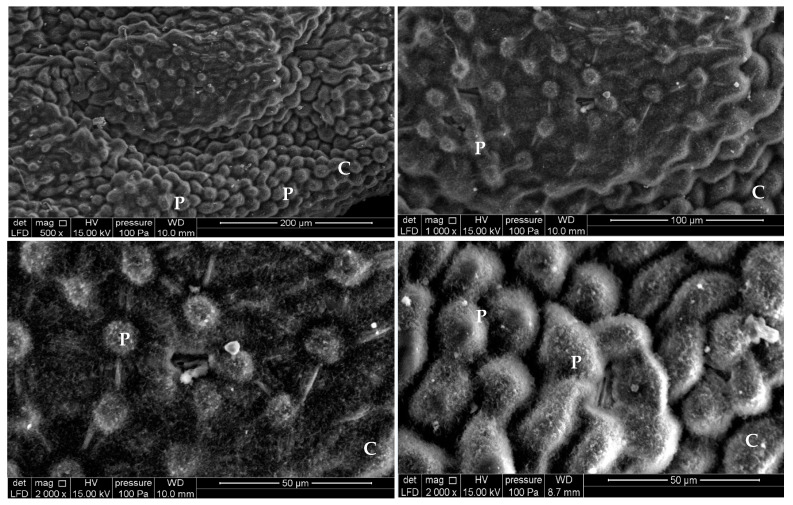
Scanning Electron Microscope micrographs of trichome from untreated leaves of *Ruta montana* (P, Peltate gland; NG, non-glandular; C, Distribution of trichomes on the leaf).

**Figure 2:**
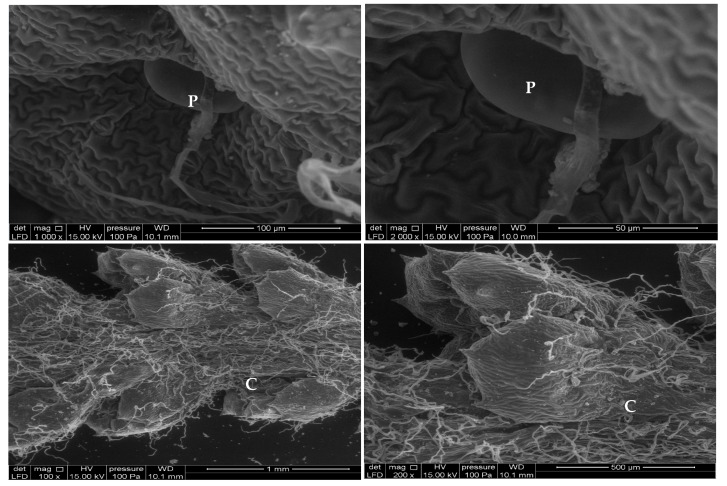
Scanning Electron Microscope micrographs of trichome leaf from *Achillea odorata* subsp. *pectinata* (P, Peltate gland; C, Distribution of trichomes on the leaf).

*Achillea odorata* subsp. *pectinata* and *Ruta montana* were collected at the flowering stage from Taza region (34°13.367’N, 003°53.111’W and 34°31.050’N, 003°58.991’W, respectively) in 2016. Plants were identified in the laboratory of Natural Resources and Environment, Polidisciplinary Faculty of Taza, Sidi Mohamed Ben Abdellah-Fez University, where samples (FPT-LRNE 33 and FPT-LRNE 34, respectively) have been deposited. Aerial parts were dried at 25 °C, then the observation of the aerial part sections was performed, to check the presence of structures responsible for essential oils, using a scanning electron microscopy (Brand: FEI Company, Model: Quanta 200 equipped with an EDAX probe for micro-analysis) (Figure 1 and Figure 2). Moreover, 100 g of each aerial part was extracted using hydro-distillation Clevenger-type apparatus for 4 h. The essential oils were stored at 4 °C until use.

### 2.2. Gas Chromatography–Mass Spectrometry (GC–MS) Analysis

Volatile compounds of AO_p_EO and RMEO were analyzed on Hewlett Packard model HP6890 gas chromatograph (Agilent Technologies, Palo Alto, CA, USA) equipped with DB-5MS capillary column (30 m × 0.25 mm i.d., film thickness 0.25 μm; Agilent Technologies, Santa Clara, CA, USA) and coupled to an HP model 5973 mass selective detector. The oven temperature was initially held at 50 °C and then increased by 7 °C/min to 300 °C. The injector temperature was 290 °C. Purified helium was used as the carrier gas with a flow rate 1mL/min, and the split ratio was 60:1. Mass spectra were obtained in EI mode at 60 eV ionization energy, and the mass range was from *m/z* 35 to 400. For each essential oil (EO), a sample of 10 μL was diluted in 990 μL of pure hexane, and 1 μL was injected for the analysis. The device was managed by a computer system type "HP Chem Station Software" G1701BA version B.01.00 and the data reworks was done with the same software [56]. The identification of each compound was based on the comparison of its retention index (RI) (calculated using n-alkanes series between C9 and C31) and its mass spectra (MS) spectra with those described in the literature [57], and by computer matching with standard reference databases (NIST98, Wiley275 and CNRS libraries).

### 2.3. Antioxidant Activities

#### 2.3.1. Free Radical Scavenging Activity by DPPH

The evaluation of the radical scavenging effect of EOs was performed using the radical 2.2-diphenyl-1-picrylhydrazyl (DPPH) method as reported by Huang et al. [58]. First, the DPPH solution (0.2 mM) was prepared in methanol. Then 2.5 mL of test samples at different concentrations (2.5–100 μg/mL) were added to 0.5 mL of DPPH solution. The absorbance was measured at 517 nm after 30 min. Ascorbic acid and Trolox were used as standard antioxidants.

The antioxidant activity was calculated using the following formula Equation (1):

DPPH scavenging activity (%) = [(A_0_ − A_s_)/Abs_0_] × 100 (1)

where A_0_ is the Absorbance of the negative control, and A_s_ is Absorbance of the test sample at 30 min. The tests were done in triplicate and the half maximal inhibitory concentration (IC_50_) values were reported as means ± SD.

#### 2.3.2. Reducing Power Assay

The reducing power activity of EOs was evaluated according to Oyaizu [59]: The mixture made up by the sample (1 mL), the phosphate buffer (2.5 mL, 0.2 M, pH 6.6) and the potassium ferricyanide (2.5 mL) was prepared. A volume of 2.5 mL of trichloroacetic acid (10%) was added after incubation for 20 min at 50 °C (water bath). Then, the solution was centrifuged at 3000 rpm/min for 10 min. Afterwards, 2.5 mL of the supernatant was mixed with 0.5 mL of 0.1% ferric chloride and 2.5 mL of distilled water. Absorbance was measured at 700 nm. The reducing power is expressed in milligram equivalence of ascorbic acid per gram of essential oil (mg AAE/g of EO).

### 2.4. Antimicrobial Activity

#### 2.4.1. Microorganisms and Growth Conditions

Food-borne bacteria tested for antimicrobial activity included Gram-positive bacteria (*Staphylococcus aureus* CECT 976, *Bacillus subtilis* DSM 6633, and *Listeria innocua* CECT 4030), Gram-negative bacteria [*Escherichia coli K12*, *Pseudomonas aeruginosa CECT 118* and *Proteus mirabilis* (National Institute of Hygiene, Rabat, Morocco: NIH)], and the yeast *Candida albicans ATCC 10231*. Bacterial strains were cultured in Mueller–Hinton Agar (MHA) or Mueller–Hinton Broth (MHB) at 37 °C. From frozen stocks (−80 °C in 20% glycerol), a pre-culture step was carried out in 1 mL of MHB at 37 °C for 5 h to aid bacteria growth. Then, 100 µL of the inoculum was spread onto MHA medium and incubated for 18h in order to detect possible airborne contaminants which may have been introduced during the opening of the tube. The next day, a colony was picked from the MHA medium and used to inoculate a 5mL MHB, then incubated at 37 °C for 18h [60]. The same steps were performed for *Candida albicans ATCC 10231*, which was cultured on Yeast Peptone Glucose Agar (YPGA) medium (5g yeast extract, 5 g Peptone, 10 g Glucose, 15–18 g Agar, in 1 liter) or Yeast Peptone Glucose (YPG) Broth medium, and incubated for 48 h at 30 °C. Cell suspensions were adjusted to 10^6^ CFU/mL for bacteria and 10^5^ spores/mL for yeast before the experiences.

#### 2.4.2. Agar Disc Diffusion Method

Antimicrobial activity was performed by the disc diffusion technique according to Rota et al. [61], with many modifications. First, sterile disks (6 mm diameter) containing 12.5 µL of pure essential oil were applied onto the surface of the MHA, which was previously spread with the test inoculum concentrations. Gentamicin (15 μg), Vancomicym (30 µg), and Amphotericin (10 μg) served as a positive control, and 10% dimethylsulfoxide (DMSO) as negative control. After the incubation, the antimicrobial effect was assessed by calculating the diameter of inhibition zones. Tests were conducted in triplicate.

#### 2.4.3. Minimum Inhibitory Concentration and Minimum Bactericidal Concentration

Minimum Inhibitory Concentrations (MICs) were realized in sterile 96 well microplates as described by Güllüce et al. [1]. First, 100 μL of MHB was distributed in all test wells, except the first well which contained 200 μL of the essential oil (25 mg/mL). A series of concentrations varying from 0.097 to 25 mg/mL were prepared by the transfer of 100 μL by serial dilutions from the first to the ninth well. Then, except for the 10th well used as sterility control, a volume of 10 μL from each well was eliminated and replaced with the test inoculum concentrations as described above. The 11th well was used as positive growth control containing only broth medium. The last well, comprising 10% DMSO (v/v), served as negative control. Then, the microplates were incubated at conditions of growth as described above. After incubation, a volume of 25 μL of an indicator of microorganism’s growth was added in each well: tetrazolium [MTT: 3-(4,5-dimethythiazol)-2-yl-2, 5-diphenyltetrazolium bromide (Sigma-Aldrich, Darmstadt, Germany) (0.5 mg/mL in sterile distilled water). The microplate was re-incubated for 30 min at 25 °C or 37 °C. Where bacterial growth was inhibited, the solution kept the initial color of MTT. To conclude, the minimum bactericidal concentration (MBC) value, 10 μL of broth from the uncolored wells, was inoculated and incubated at growth conditions.

### 2.5. Antibacterial Activity of Essential Oils in Cabbage and Barley Food Model Systems

#### 2.5.1. Preparation of Model Food Systems

Preservative activity of AOpEO showing high antimicrobial potency was tested using two food model systems according to Catherine et al. [8]. Cabbage bought from the local supermarket was cut into fine pieces and mixed with distilled water (1:2, *w*/*v*). The pH of the juice was adjusted to 7.2. Barley soup was prepared by mixing barley powder with distilled water (10%, *w*/*v*) (pH: 5.6). Thereafter, a volume of 50 mL of each food model system was introduced separately into bottles of 250 mL and sterilized. After cooling, bottles (cabbage and barley food systems) were divided into three groups: the first group received a value of the MIC concentration of AOpEO, the second group received a value of 4× MIC, and the third group served as control (without AOpEO). Food model systems were inoculated with 10^6^ CFU/mL of *Bacillus subtilis DSM 6633* and *Proteus mirabilis NIH* and incubated at 37 °C for 28 days. Experiments were done in triplicates.

#### 2.5.2. Bacterial Analysis

*Bacillus subtilis DSM 6633* and *Proteus mirabilis NIH* strains were counted on Plate Count Agar medium during storage period from the 1st to the 28th day. The results are expressed in log CFU/mL.

### 2.6. Statistical Analysis

All tests were done in triplicates. Values of each experiment were expressed as mean ± standard deviation (SD) and were subjected to analysis of variance (one-way ANOVA). The statistical analysis was performed using GraphPad Prism version 6.00 (GraphPad Inc., San Diego, CA, USA). Differences (between groups) were considered as statistically significant at *p* < 0.05.

## 3. Results and Discussion

### 3.1. Chemical Composition

**Figure 3:**
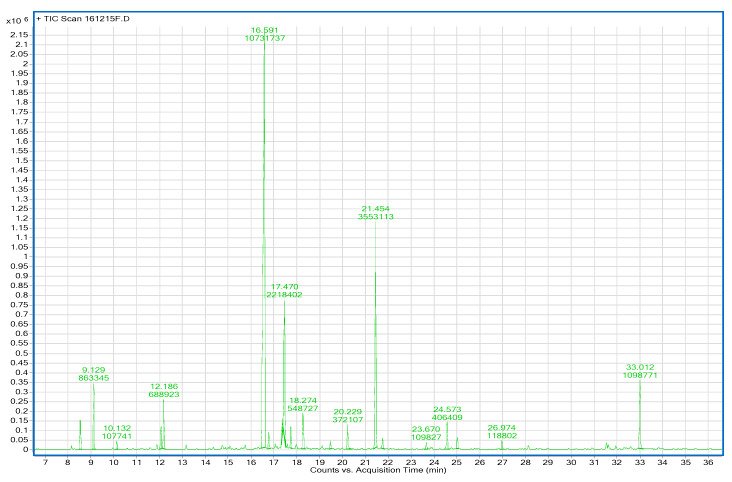
Chromatogram of gas chromatography analysis of *Achillea odorata* subsp. *pectinata* essential oil.

**Figure 4:**
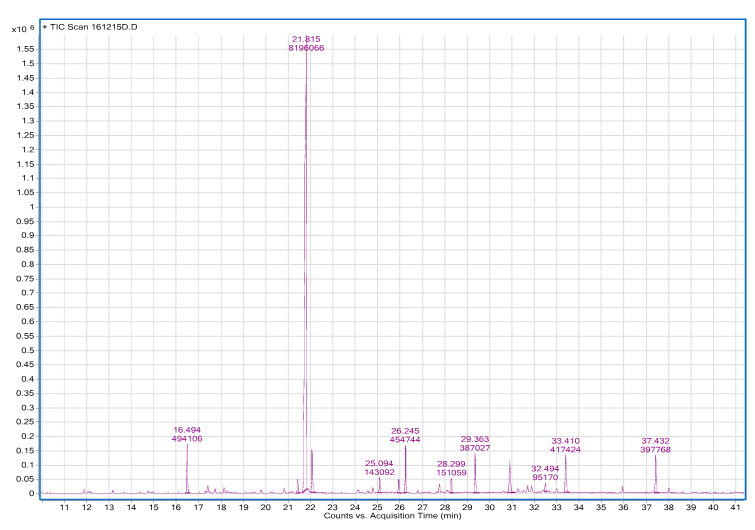
Chromatogram of gas chromatography analysis of *Ruta montana* essential oil.

Essential oil yields (*w*/*w*) were 1.04% ± 0.01% and 0.37% ± 0.03% for AO_p_EO and RMEO, respectively. The results of gas chromatography analysis of *Achillea odorata* subsp. *pectinata* and *Ruta montana* are shown in Figure 3 and Figure 4. GC analysis was coupled with mass spectrometry to identify volatile compounds produced by both plants. Results of GC-MS analysis of the essential oils are listed in Table 1. In total, 21 and 25 compounds were identified in AO_p_EO and RMEO, respectively. The results showed that the oxygenated monoterpenes were the main components of AO_p_EO, which were dominated by camphor (45.01%), bornyl acetate (15.07%), followed by borneol (11.33%), and 1.8-cineole (eucalyptol) (2.96%). For RMEO, the essential oil was characterized by a rich presence of methylketone 2-undecanone (63.97%) as a major compound, followed by camphor (3.82%) and cyclopropanecarboxylic acid (3.66%).

To the best of our knowledge, the volatile compounds of the subspecies AO_p_EO from Morocco have not been studied. In Algeria, an analysis of the *Achillea odorata* L. subsp. *pectinata* (Lamk) var. *microphylla* (Willd.) Willk. showed that, in the flowering period, the major compound is camphor, with a percentage of 22.9% to 26.3%, followed by 1,8-cineole (15.7% to 17.8%) and then the *α* -pinene (11.3% to 12.5%) [62]. In addition, the compounds bornyl acetate (15.07%) and borneol (11.33%) are identified in the essential oil of the Moroccan subspecies, whereas they are absent in the volatile compounds content of the essential oil of the Algerian variety. It was also noted that AO_p_EO is richer in 1,8-cineole (15.7% to 17.8%) and *α*-pinene (11.3% to 12.5%) in comparison with their content in the essential oil of our subspecies. As described above, the *Achillea* species is dominated by oxygenated monoterpenes, meaning that our results were in agreement with previous research.

For RMEO, the results are similar to other studies reporting that 2-undecanone is the major compound of RMEO species with different percentages. Indeed, Kambouche et al. [63] identified the presence of 2-undecanone with a percentage of 32.8%. Belkassam et al. [64] and Boutoumi et al. [65] found that this product is present at 60.19% and 67%–67.4%, respectively, in RMEO.

The qualitative and quantitative variations encountered in the volatile compounds of the essential oils of AO_p_EO and RMEO may be due to many factors, such as the environment, harvest season, ecological parameters, and also the extraction methods used, as well as the trial conditions [66,67,68,69,70].

### 3.2. Antioxidant Activity

The antioxidant activity of AO_p_EO and RMEO was examined by DPPH and reducing power tests. The obtained results are summarized in Table 2. The results demonstrated that AO_p_EO has a higher capacity to reduce the DPPH (IC_50_ = 189.8 ± 1.09 µg/mL) than RMEO (IC_50_ = 244.62 ± 0.34 µg/mL), but they were all less potent than the standards used as positive controls, namely Trolox and ascorbic acid, IC_50_ = 1.4 ± 0.04 µg/mL and IC_50_ = 1.82 ± 0.025 µg/mL, respectively (statistically significant at *p* < 0.05).

In the reducing power test, in which results are expressed in milligram equivalence of ascorbic acid per gram of extract (mg AAE/g EO), the highest reducing power was exhibited by RMEO, as 1.39 ± 0.07 mg AAE/g of EO, while that of AO_p_EO was 0.85 ± 0.24 mg AAE/g of EO.

The difference in antioxidant capacities between AO_p_EO and RMEO may be due to the variability in chemical composition [71,72]. However, variations in the antioxidant effects of essential oils, tested by DPPH and FRAP assay, may be due to the differences in reagents used by each method [73]. Indeed, the DPPH assay evaluates the capacity of essential oils to scavenge free radicals, while the FRAP method assesses EO’s reducing power. Oxidative degradation can occur in food matrices during storage; specifically, the lipid peroxidation, which is a major cause of food deterioration, and which affects its organoleptic qualities [74,75]. Thus, the interest in the use of essential oils as food preservatives for increasing the food shelf life is related to their efficacy in scavenging the reactive oxygen species (ROS) [76,77].

### 3.3. Antimicrobial Activity

In vitro tests of the antimicrobial activity of AO_p_EO and RMEO, by using the filter paper disc diffusion and the microdilution methods against microorganism tests, are summarized in Table 3 and Table 4. The obtained results revealed a sensitivity variation between the microorganisms tested.

Among the Gram-positive bacteria, *Bacillus subtilis DSM 6633* was the most sensitive strain to the AO_p_EO and RMEO, with an inhibition zone of 31 ± 1 mm and 21.33 ± 1.52 mm, respectively. The MBC/MIC values indicate that both oils exhibit a bacteriostatic effect against *B.subtilis,* while *E. coli K12* was the most resistant, with inhibition zones of 6.00 ± 0.00 mm–9.33 ± 1.52 mm. Moreover, *S. aureus* (12 ± 1.52 mm–12 ± 1 mm) and *L. innocua* (12 ± 1 mm–10.33 ± 1.52 mm) were less sensitive to AO_p_EO and RMEO, respectively.

Concerning Gram-negative bacteria, the strain most sensitive to EOs was *P. mirabilis NIH* (30.33 ± 2.08 mm–16.66 ± 1.15 mm). Moreover, The MBC/MIC values mean that AO_p_EO and RMEO exhibit bactericidal and bacteriostatic effects, respectively. An important antifungal activity was observed against *C. albicans* (25.33 ± 0.57 mm–21.66 ± 0.57 mm), with a fungicidal effect exhibited by AO_p_EO.

There are no reports on the antimicrobial activity of AO_p_EO, and the unique study on the antimicrobial activity of *Achillea odorata* L. subsp. *pectinata* (Lamk) var. *microphylla* (Willd.) Willk against *P. aeruginosa, E.coli, S.aureus, E.faecalis, C. herbarum, A. fumigatus, F. oxysporum,* and *A. flavus* reported that the bacterial strain’s inhibition zone values are in the range of 6 to 17 mm [62], and that the antifungal effects on *A. alternaria, A. fumigatus* and *C. herbarum* have MIC values of 4 µL/mL and 5 µL/mL, respectively. Furthermore, previous investigations into the antimicrobial activity of many *Achillea* species’ essential oils, against Gram-positive and Gram-negative bacteria and fungi, report their important efficacy in inhibition of the microorganisms tested [78,79,80,81,82,83].

Regarding the antimicrobial activity of *Ruta montana*’s essential oil, Mohammedi et al. [84] reported a moderate antimicrobial effect against eight microbial species, including *B. subtilis, S. aureus, E.coli, P. aeruginosa* and *C. albicans* tested in our study (9.2 ± 0.5 mm ≤ inhibition zones’ diameters ≤ 18 mm). In another work, Djarri et al. [85] indicated that the *R. montana*’s essential oil exhibits a good antibacterial activity against *E. coli, K. pneumoniae P. aeruginosa,* and *S. aureus*, with an MIC value of 20–80 µg/mL. In addition, an important antifungal activity of the oil (1000 µg/disk) was revealed against *B.cinerea*, *F. solani*, *F. oxysporum* and *A.oryzae* (MIC = 100, 140, 160 and 1100 µg/mL, respectively) [86].

From the point of view of the susceptibility of Gram-negative and Gram-positive organisms, it has been demonstrated that the Gram-negative bacteria are less sensitive to plant extracts than Gram-positive bacteria [87,88], since Gram-negative bacteria possess double membranes which protect them against the antibacterial agents [89,90]. The present work showed, on the one hand, that RMEO is more active against Gram-positive bacteria. This activity could be due to the presence of 2-undecanone and 2-undecanol, known for their antimicrobial activity [91,92]. On the other hand, AO_p_EO was active against both Gram-positive (*B. subtilis*) and Gram-negative (*P. mirabilis*) bacteria; this non-selective antibacterial activity is associated with the membrane composition differences of microorganism tested. These findings may be related to the presence of a high content of camphor, bornyl acetate and borneol. Indeed, the antibacterial and antifungal activities of these compounds have been demonstrated in earlier works [93,94,95,96,97,98,99]

### 3.4. Antibacterial Effect of Essential Oils in Food Model Systems

**Figure 5:**
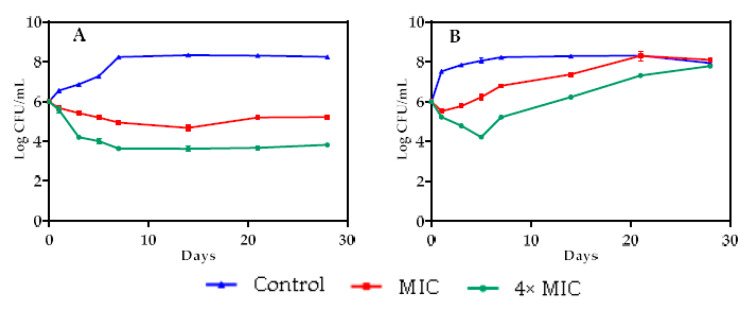
Effect of MIC and 4× MIC of *Achillea odorata* subsp. *pectinata* essential oil on *Proteus mirabilis* (**A**) and *Bacillus subtilis* (**B**) in cabbage food system.

**Figure 6:**
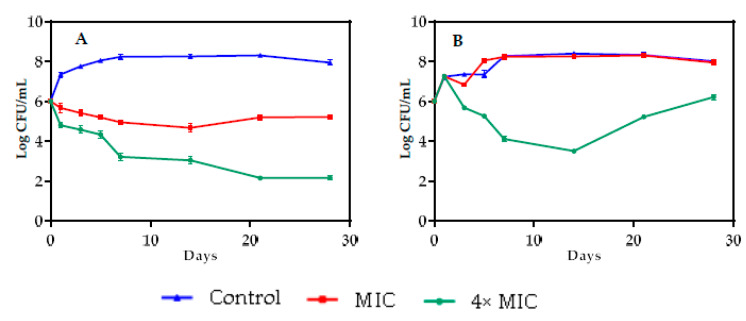
Effect of MIC and 4× MIC of *Achillea odorata* subsp. *pectinata* essential oil *on Proteus mirabilis* (**A**) and *Bacillus subtilis* (**B**) in Barley food system.

The antibacterial activity of *Achillea odorata* subsp. *pectinata* oil in model food systems was assessed, with *B. subtilis* as the Gram-positive bacteria and *P. mirabilis* as the Gram negative bacteria, separately. *Achillea odorata* subsp. *pectinata* oil was effective in reducing bacterial count in food model systems, cabbage and barley, during storage (Figure 5 and Figure 6). The reduction was dose-dependent.

In cabbage system, the reduction of the count of *P. mirabilis* strain by 4× MIC of AO_p_EO was significant (*p* < 0.05), as compared to the control, from the 1st day to the end of storage duration (10^4^ CFU/mL), while MIC stabilized the growth of *P. mirabilis* at 10^5^ CFU/mL up to the 28th day (Figure 5A). However, for *B. subtilis*, a significant reduction (*p* < 0.05) was exhibited by MIC of AO_p_EO on the first day, and 4× MIC of AO_p_EO up to the fifth day (10^4^ CFU/mL), when the bacteria returned to normal growth (Figure 5B).

In barley systems, a significant (*p* < 0.05) inhibition of *P. mirabilis* growth was observed at 4× MIC of AO_p_EO, as compared to the control, up to the 28th day. At MIC of AO_p_EO_,_ the growth of *P. mirabilis* was stabilized at 10^5^ CFU/mL up to the 28th day (Figure 6A). *B. subtilis* barley systems showed that 4× MIC of AO_p_EO reduced significantly (*p* < 0.05) the growth of this strain in the first 14 days (10^4^ CFU/mL), then the bacteria return to normal growth. No effect on *B. subtilis* growth was observed after the addition of the MIC of AO_p_EO (Figure 6B).

Our findings revealed, for the first time, the long-term effectiveness (up to 28 days) of AOpEO against *P. mirabilis* in food model system. Few studies reported the same effectiveness of other EOs against food-born bacteria [4,8]. Like in in vitro tests, *P. mirabilis* was more sensitive to AOpEO as compared to *B. subtilis* in the food model system. In fact, AOpEO only exhibited a bacteriostatic effect against P. mirabilis in the food model system, while it showed a bactericidal effect in vitro, whereas AOpEO lost its bacteriostatic effect against *B. subtilis*, suggesting that after a few days *B. subtilis* could develop a resistance to AOpEO, while *P. mirabilis* remains sensitive up to 28 days. On the other hand, the decrease of effectiveness of the antibacterial effect of EOs in food systems, as compared to in vitro tests, could be due to certain factors. According to Burt et al. [4], at low pH value, the hydrophobicity of EOs increases, easing their penetration into the target cell, indicating that the pH of the medium could alter the antibacterial activity in the food system. Mejholm and Dalgaard [100] showed that the affinity of essential oils to fatty acids decreases their interaction with bacteria in the aqueous phase. Another suggestion is that the food system is a highly nutritional environment that can allow the regrowth of food-borne strains that have been damaged [101]. To overcome these problems, many researchers have suggested that it is mandatory to add higher concentrations (of around 10- to 100-fold of MIC) of an essential oil in food model systems [102,103,104].

## 4. Conclusions

AO_p_EO and RMEO collected from the Taza region (northern Morocco) showed a variation in chemical compositions, antioxidant and antimicrobial activities: AO_p_EO is rich with camphor, bornyl acetate and borneol, while RMEO is characterized by a dominance of 2-undecanone. A significant capacity of AO_p_EO to reduce the DPPH was observed, while the highest reducing power was exhibited by RMEO. The strongest antibacterial activity against *B. subtilis* and *P. mirabilis* strains was obtained for AO_p_EO. Due to their high antibacterial activity, *B. subtilis* and *P. mirabilis* were exposed long-term to AOpEO in food model systems, which showed a high efficacy against *P. mirabilis* that was maintained up to 28 days. Rare studies have reported a similar long-term maintenance of EOs’ antibacterial effects in food model systems. This advantage makes AOpEO very useful as a food preservative agent at an industrial level. Future studies should be done into the influence of essential oil addition on sensory and textural properties of foods, as well as toxic effects, before any application at an industrial level.

## Figures and Tables

**Table 1:** Chemical composition of essential oils obtained from *A. odorata* subsp. *pectinata* and *R. montana.*

Compounds	RI *	*A. Odorata* Subsp. *Pectinata*	*R. Montana*
		Area %	
*α*-Pinene	930	1.61	nd
Camphene	947	3.58	nd
*β-* Pinene	975	0.45	nd
Paracymene	1025	nd	0.31
Limonene	1030	1.42	nd
1,8-Cineole (Eucalyptol)	1034	2.96	nd
Camphor	1153/1151	45.01	3.82
2(1H)–pyridinone	1158	1.07	-
Bicyclo [2.2.1] heptan-3-one	1165	0.35	-
Cyclopentene,3-ethylidene-1-methyl	1172	2.05	-
Borneol	1175/1173	11.33	0.66
Terpineol-4	1181	1.44	nd
Decanone-2	1191	nd	0.46
Terpinolene	1195	2.3	nd
Cyclopentane, 2-methyl-1-methylene	1228	0.59	nd
Geranylbromide	1249	1.56	nd
Geranial	1266	nd	0.42
Bornyl acetate	1283	15.07	1.16
Phenol,2-(2-methylpropyl)	1292	0.72	-
2-undecanone	1294	nd	63.97
2-undecanol	1302	nd	3.25
Eugenol	1349	0.47	nd
Nerol	1376	1.69	nd
Trimethyl-tetrahydronaphtalene	1383	nd	0.45
*Cis*-Jasmone	1390	0.82	nd
Dodecacone-2	1392	nd	1.14
*β-Trans*-caryophyllene	1419	nd	1.1
2-Acetoxydodecane	1428	nd	3.66
*β-E*- Farnesene	1451	0.49	nd
Tridecanone-2	1493	nd	1.2
Tetramethylsuccinimide	1529	nd	3
Caryophyllene oxide	1581	nd	3.38
*Ɣ*-Gurjunene	1593	nd	0.42
(-)-isoledene	1608	nd	0.73
2-pentene, 4-methyl	1614	nd	0.65
Adamantane	1636	nd	0.78
Globulol	1653	nd	0.4
*β*-Eudesmol	1654	4.74	nd
3-Heptene,7-phenyl	1668	nd	3.38
2-nonen-4-one	1761	nd	0.59
1,3-benzodioxole,5-(2,2-dimethyl)	1820	nd	3.09
Trimethyl-6,10,14-pentadecanone-2	1841	nd	0.41
Isomaturnin	2162	nd	0.44
Total		99.72	98.87

* RI: identification by Kovats indices. Retention index relative to C9–C31 on DB-5 MS capillary column. nd: not detected.

**Table 2:** Antioxidant activity of *A. odorata* subsp. *pectinata* and *R. montana* essential oils.

Assays	Essential Oils		Ascorbic Acid	Trolox
	*A. Odorata* Subsp. *Pectinata*	*R. Montana*		
DPPH (IC_50_, µg/mL) *	189.8 ± 1.09 ^a^	244.62 ± 0.34 ^b^	1.82 ± 0.025 ^c^	1.4 ± 0.04 ^d^
Reducing power (mg AAE/g of EO) **	0.85 ± 0.24	1.39 ± 0.07	ND	ND

Values represent means (standard deviations) for triplicate experiments; values with different superscripts (a–d) were significantly different at *p* < 0.05. * IC_50_: the concentration at 50% of inhibition. ** mg AAE/g EO: milligram equivalence of ascorbic acid per gram of essential oil; ND: not determined.

**Table 3:** Antimicrobial activity of *A. odorata* subsp. *pectinata* and *R. montana* essential oils determined by disc diffusion method.

	Inhibition Zones Diameter (mm) *					
	Essential Oils			Standard Antimicrobial		
	*A. Odorata* Subsp. *Pectinata*	*R. Montana*	Gentamicin (15 μg)		Vancomicym (30 µg)	Amphotericin (10 μg)
*S. aureus CECT 976*	12 ±1.52 ^a^	12 ± 1 ^a^	34.33 ± 0.57 ^b^		30.66 ± 0.57 ^c^	NT
*B. subtilis DSM 6633*	31 ± 1 ^a^	21.33 ± 1.52 ^b^	26 ± 1 ^c^		27.66 ± 0,57 ^d^	NT
*L. innocua CECT 4030*	12 ± 1 ^a^	10.33 ± 1.52 ^a^	17.66 ± 0.57 ^b^		25.33 ± 0.57 ^c^	NT
*E. coli K12*	9.33 ± 1.52 ^a^	6 ± 0.00 ^b^	20.33 ± 0.5 ^c^		8 ± 0.00 ^ab^	NT
*P. aeruginosa CECT 118*	12 ± 1 ^a^	9 ± 2.64 ^b^	19 ± 1 ^c^		6 ± 0.00 ^b^	NT
*P. mirabilis NIH*	30.33 ± 2.08 ^a^	16.66 ± 1.15 ^b^	28.66 ± 0.57 ^a^		24.33 ± 0.57 ^c^	NT
*C. albicans ATCC 10231*	25.33 ± 0.57 ^a^	21.66 ± 0.57 ^b^	NT		NT	18.66±1.15^c^

* The diameter of the inhibition zones (mm), including diameter of disc 6 mm, are given as mean ± SD of triplicate experiments; NT: not tested; Within each line, Different letters (a–c) indicate significant differences (*p* < 0.05).

**Table 4:** The Minimum Inhibitory Concentration (MIC) and Minimum Bactericidal Concentration (MBC) (mg/mL) of *A. odorata* subsp. *pectinata* and *R. montana* essential oils.

Bacterial Strains	Essential Oils				
	*A.* Subsp. *Pectinata*			*R. Montana*	
	MIC	MBC	MIC		MBC
*S. aureus CECT 976*	12.5	25	>25		>25
*B. subtilis DSM 6633*	0.19	3.12	0.39		6.25
*L. innocua CECT 4030*	25	>25	nt		NT
*P. aeruginosa CECT 118*	25	>25	nt		NT
*P. mirabilis NIH*	0.19	0.19	0.78		6.25
*C. albicans ATCC 10231*	6.25	12.5	6.25		>25

MIC: minimum inhibitory concentration; MBC: minimum bactericidal concentration; NT: not tested.
